# The first clawed lobster virus *Homarus gammarus* nudivirus (HgNV n. sp.) expands the diversity of the *Nudiviridae*

**DOI:** 10.1038/s41598-019-46008-y

**Published:** 2019-07-12

**Authors:** Corey C. Holt, Michelle Stone, David Bass, Kelly S. Bateman, Ronny van Aerle, Carly L. Daniels, Mark van der Giezen, Stuart H. Ross, Chantelle Hooper, Grant D. Stentiford

**Affiliations:** 1 International Centre of Excellence for Aquatic Animal Health Theme, Centre for Environment, Fisheries and Aquaculture Science (Cefas), Barrack Road, Weymouth, Dorset DT4 8UB United Kingdom; 2 Biosciences, University of Exeter, Stocker Road, Exeter, EX4 4QD United Kingdom; 3 The National Lobster Hatchery, South Quay, Padstow, PL28 8BL United Kingdom; 4 Centre for Sustainable Aquaculture Futures, University of Exeter, Stocker Road, Exeter, EX4 4QD United Kingdom; 5 Department of Life Sciences, The Natural History Museum, Cromwell Road, Kensington, London SW7 5BD United Kingdom

**Keywords:** Pathogens, Metagenomics

## Abstract

Viral diseases of crustaceans are increasingly recognised as challenges to shellfish farms and fisheries. Here we describe the first naturally-occurring virus reported in any clawed lobster species. Hypertrophied nuclei with emarginated chromatin, characteristic histopathological lesions of DNA virus infection, were observed within the hepatopancreatic epithelial cells of juvenile European lobsters (*Homarus gammarus*). Transmission electron microscopy revealed infection with a bacilliform virus containing a rod shaped nucleocapsid enveloped in an elliptical membrane. Assembly of PCR-free shotgun metagenomic sequencing produced a circular genome of 107,063 bp containing 97 open reading frames, the majority of which share sequence similarity with a virus infecting the black tiger shrimp: *Penaeus monodon* nudivirus (PmNV). Multiple phylogenetic analyses confirm the new virus to be a novel member of the Nudiviridae: *Homarus gammarus* nudivirus (HgNV). Evidence of occlusion body formation, characteristic of PmNV and its closest relatives, was not observed, questioning the horizontal transmission strategy of HgNV outside of the host. We discuss the potential impacts of HgNV on juvenile lobster growth and mortality and present HgNV-specific primers to serve as a diagnostic tool for monitoring the virus in wild and farmed lobster stocks.

## Introduction

Viral pathogens and the diseases that they impart are a particularly significant source of production loss in the cultivation of crustaceans^1^. Despite the preponderance of known viruses in a wide range of crustacean hosts and their ubiquity in the aquatic environment, with abundance estimates of ~10^8^ viruses per ml of productive coastal waters^2^, there have been no reported examples of naturally-occurring viruses infecting any clawed-lobster species (Decapoda; Nephropidae).

The currently unclassified *Panulirus argus* virus 1 (PaV1) is so far the only virus described from lobsters, in this case infecting mesodermal cells of the Caribbean spiny lobster (*Panulirus argus)* in Florida and throughout the Caribbean^3^. The infection, characterised by a milky colouration of the haemolymph and lethargy of the host, initially infects fixed phagocytes in the hepatopancreas (HP), prior to spreading to cells of the connective tissues. Since its initial discovery in juvenile life stages in the United States, PaV1 has been found in wild and cultured host populations throughout the Caribbean^4^. Remarkably, healthy lobsters demonstrate avoidance behaviours towards those infected with the PaV1 virus^5^. As with several other invertebrate pathogens, mortality rate is higher in animals with a carapace length of less than 16 mm^6^. Until now, there have been no reports of PaV1-like viruses or any other viruses in any species within the clawed lobster genera of the family Nephropidae. In experimental conditions, White spot syndrome virus (WSSV), a double stranded DNA (dsDNA) virus of the family *Nimaviridae*, has been demonstrated to infect (and cause disease in) both American lobster *(Homarus americanus*)^7^ and the European lobster (*H*. *gammarus*)^8^ as well as numerous other decapod crustacean taxa^9^. However, WSSV has not been detected in wild or cultured Nephropidae.

Viral diseases have led to substantial bottlenecks to shrimp aquaculture production. Monodon baculovirus (MBV), the causative agent of spherical baculovirosis, was the first virus reported in penaeid shrimp^10^. Phylogenetic analyses and genomic reconstruction has since suggested that MBV be reclassified as *Penaeus monodon* nudivirus (PmNV) and be reassigned to the *Nudiviridae*^11,12^, a family of dsDNA viruses which to that point was exclusively comprised of viruses infecting insects. Although initially named to reflect a lack of occlusion body formation (large protein lattices which protect the bacilliform-shaped virions and facilitate transmission outside of the host), there are now multiple examples within the *Nudiviridae* where occlusion bodies have been observed, or where sequence and structural homologs of the *polyhedrin* gene have been found within the genome^12–14^. Seven fully sequenced virus species have been characterised as nudiviruses: *Penaeus monodon* nudivirus (PmNV)^12^; *Gryllus bimaculatus* nudivirus (GbNV), infecting the nymph and adult stages of several cricket species^15^; *Heliothis zea* nudivirus-1 (HzNV-1), a persistent pathogen of insect cell lines^14^; *Helicoverpa (*syn. *Heliothis) zea* nudivirus-2 (HzNV-2), the sexually transmitted corn earworm moth virus which can cause sterility in the host^16^; *Oryctes rhinoceros* nudivirus (OrNV), a biological control agent used to manage palm rhinoceros beetle populations^17^; *Tipula oleracea* nudivirus (ToNV) a causative agent of nucleopolydreosis in crane fly larvae^13^; and *Drosophila innubila* nudivirus (DiNV)^18,19^, which causes significant reductions to fecundity and lifespan^18^. Three further viruses isolated from metagenomic sequencing of *Drosophila melanogaster* (Kallithea virus^20^, Tomelloso virus and Esparto virus) have also been described as nudiviruses (Table 1). However, the genomes of these three *Drosophila* viruses have yet to be analysed with respect to their phylogenetic position. There is also evidence of ancestral nudivirus integration into the host genome (*Nilaparvata lugens* endogenous nudivirus *(NleNV))*^21^ and a sister group of the nudiviruses, the bracoviruses, associated with Braconid wasp hosts, where viral genes are also integrated into the host genome^22^. Finally, a large DNA virus infecting the hepatopancreas of the European brown shrimp, *Crangon crangon* has also been proposed as a putative member of the *Nudiviridae* albeit based upon limited genomic information^23,24^.

**Table 1:** Comparative genomic data of sequenced nudiviruses.

Name	Initial Host	Genome Size (bp)	Number of ORFs	Gene Density (per kb)	GC Content (%)	Reference
HgNV	European lobster (*Homarus gammarus*)	107 063	97	1.10	35.3	This study
GbNV	Field cricket (*Gryllus bimaculatus*)	96 944	98	0.99	28.0	^15^
HzNV-1	Corn earworm (*Heliothis zea*)^†^	228 089	155	1.47	41.8	^14^
HzNV-2	Corn earworm (*Heliocoverpa zea syn*. *Heliothis*)	231 621	113	2.05	41.9	^16^
OrNV	Rhinoceros beetle *(Oryctes rhinoceros)*	127 615	139	0.92	42.0	^17^
PmNV	Black tiger shrimp (*Penaeus monodon*)	119 638	115	1.04	34.5	^12^
ToNV	Crane fly (*Tipula oleracea*)	145 704	131	1.11	25.5	^13^
DiNV	Drosophilid fly *(Drosophila innubila)*	155 555	107	1.45	30.0	^19^
*KNV	Common fruit fly (*Drosophila melanogaster*)	152 388	95	1.60	38.9	KX130344
*TNV	Common fruit fly (*Drosophila melanogaster*)	122 307	93	1.32	39.6	KY457233
*ENV	Common fruit fly (*Drosophila melanogaster*)	183 261	87	2.11	29.5	KY608910

^†^Cell line. *Direct submission to GenBank - number of ORFs, gene density and GC content estimated from database entry. Accession numbers provided where journal reference of genome annotation is not available.

As part of a large UK-based lobster rearing study assessing the growth of hatchery-reared European lobsters in novel sea-based container culture (SBCC) systems (Lobster Grower, www.lobstergrower.co.uk), we conducted a histology-led health screening of a large cohort of individuals (n = 1,698), sampled at several time points throughout a multi-year production cycle. We observed a distinctive histopathology of the hepatopancreas of juvenile lobsters in both hatchery and sea container phases of production. Intranuclear inclusions appeared within the hepatopancreatocytes of affected individuals; later confirmed by transmission electron microscopy (TEM) as of viral aetiology. Genome assembly of PCR-free shotgun metagenomic sequences confirmed the presence of a novel member of the *Nudiviridae*; hereby named *Homarus gammarus* nudivirus, the first virus described infecting any clawed lobster genus. Here, we present the fully annotated genome of HgNV, comprising a single contiguous sequence, together with diagnostic primers and reference histology and ultrastructure to aid in future identification in natural and aquaculture settings. HgNV is now the second confirmed aquatic nudivirus.

## Results

### Histological sectioning reveals virus-associated pathology

**Figure 1:**
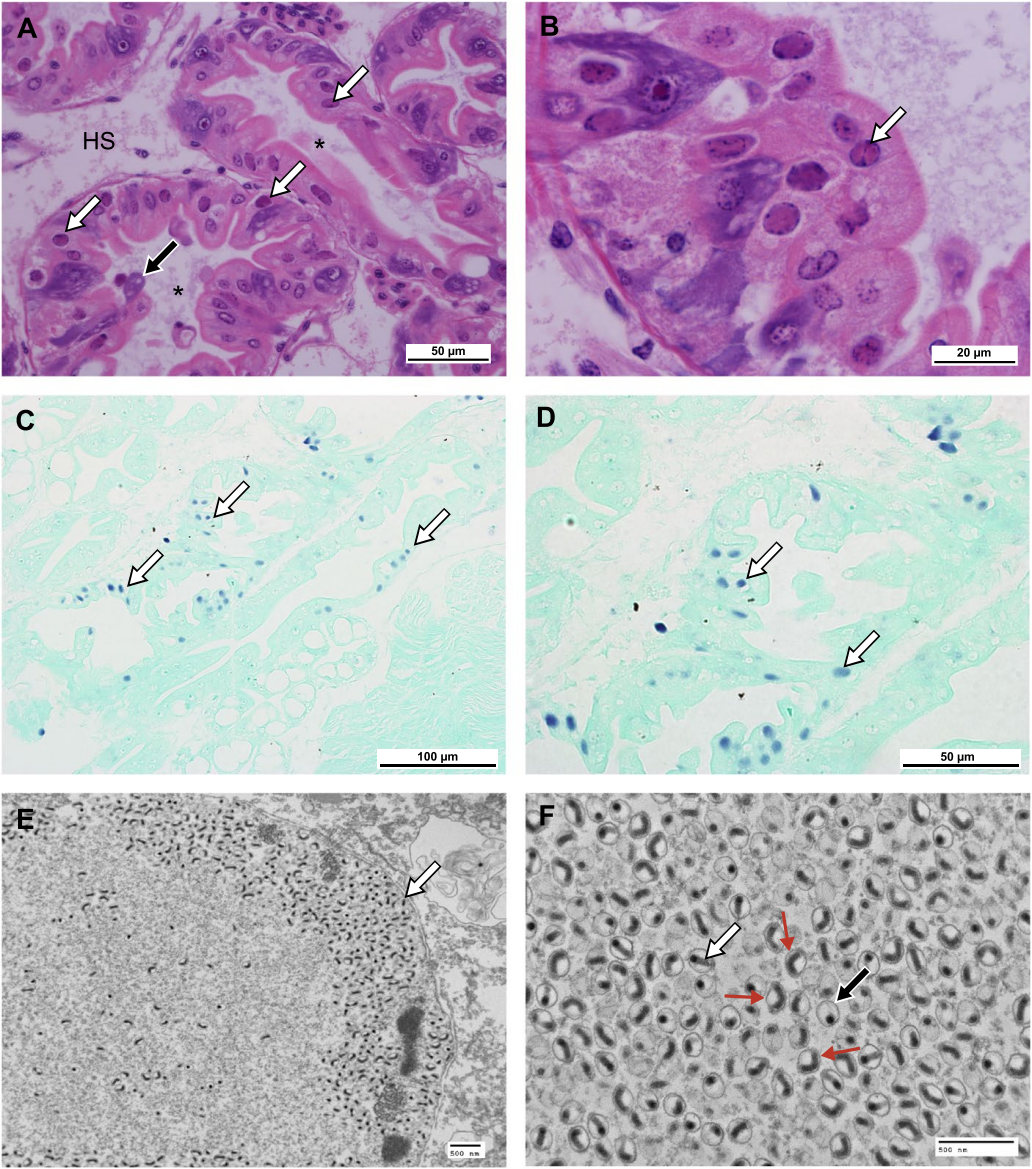
*Homarus gammarus* nudivirus (HgNV) infection within the hepatopancreas. (**A**) Section through the hepatopancreas, haemal sinus (HS) surrounds the tubules, cross section of the tubules shows a clear lumen (*). Infected nuclei within the epithelial cells of the tubules are enlarged, with emarginated chromatin and possess an eosinophilic inclusion body (white arrows). Infected cells (black arrow) may be sloughed into the lumen of the tubules. H&E Stain. Scale bar = 50 µm. (**B**) Infections can be seen within multiple epithelial cells, infected nuclei appearing larger than uninfected nuclei. Margination of the chromatin can form septa leading to the appearance of discrete intranuclear compartmentalisation (arrow). H&E Stain. Scale bar = 20 µm. (**C**,**D**) HgNV-specific *DNA polymerase* probe hybridised to infected nuclei (arrows) within epithelial cells of the hepatopancreas. *In-situ* hybridisation. Scale bar = 100 µm, 50 µm respectively. (**E**) Nucleus from a HgNV infected cell containing rod-shaped virions. Virions accumulate at the periphery of the nuclear membrane (arrow), TEM. Scale bar = 500 nm. (**F**) Longitudinal (white arrow) and transverse sections (black arrow) of HgNV virions within the nucleus. Virions possess an electron dense nucleocapsid surrounded by a trilaminar membrane (envelope). The rod shaped nucleocapsid appears to bend within the envelope forming a “u” or “v” shape in some cases (line arrows). TEM. Scale bar = 500 nm.

Lobsters did not appear to display any clinical signs of infection with HgNV. Histopathology of the virus infection was apparently limited to the tubule epithelial cells of the hepatopancreas (HP), observed in fibrilar (F) and reserve (R) cells. Infected cells contained hypertrophic nuclei occupied by a single, large eosinophilic inclusion. This inclusion displaced the host chromatin resulting in the latter’s emargination against the nuclear envelope (Fig. 1A,B). In some cases, this margination of the chromatin causes the formation of septa leading to the appearance of intranuclear compartmentalisation. Viral infection occurred either within the nuclei of isolated cells, within the closely opposing cells of a single tubule, within numerous cells of several closely opposed tubules, or generally throughout the tubules of the hepatopancreas. Often, epithelial cells containing virus-infected nuclei detached from the basement membrane of the tubule and were sloughed to the tubule lumen, presumably for excretion via the gut.

### Infection prevalence in hatchery and sea-based juvenile lobsters

**Figure 2:**
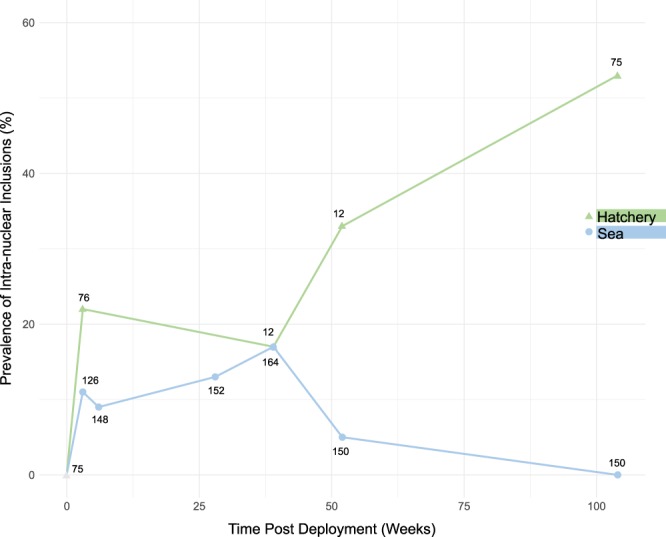
Prevalence of intranuclear inclusions in sea-based and hatchery lobsters over 104 weeks. Proportion of surviving lobsters displaying histopathological signs of viral infection. Green triangles: hatchery-based animals. Blue circles: sea-based animals. Sample size indicated at each point.

Intranuclear inclusions were observed in 12.72% of all samples processed for histology (145/1140) across the two-year sampling period. In sea-based lobsters the prevalence of intranuclear inclusions was highest at 39 weeks post deployment (17%) (Fig. 2). At this time point, the percentage of individuals displaying histological signs of viral aetiology was the same in both the hatchery and sea-based populations. However, whereas intranuclear inclusions were not evident in sea-based lobsters at 104 weeks (0%), prevalence had peaked in hatchery reared lobsters (53%) at this time (Fig. 2). Prevalence was generally observed to be higher in hatchery-based individuals compared to those retained in SBCC systems.

Three of the 150 (52 week) sea-based lobsters tested were PCR positive for HgNV, all of which were histologically positive. An additional four sea-based samples, displaying histopathological signs of intranuclear inclusions, did not amplify with HgNV-specific primers. Of the 12 hatchery-based animals tested, five were PCR positive for HgNV, three of which were histologically positive. An additional hatchery-based sample showed histological signs of nuclear infection but did not test positive with PCR. *In-situ* hybridisation of the HgNV-specific amplicon probe confirms detection and demonstrates localisation of the target HgNV DNA polymerase gene in infected tissues (Fig. 1C,D).

### Transmission electron microscopy (TEM) confirms the presence of viral infection

Transmission electron microscopy revealed the presence of masses of enveloped virions accumulated at the nuclear membrane and surrounding the virogenic stroma (Fig. 1E,F). Virions exhibited an electron-dense nucleocapsid showing a bacilliform morphology and were contained within an elliptical membrane. In some cases, the rod-shaped nucleocapsids appeared “u” or “v” shaped within the envelope (Fig. 1F). The mean length of the enveloped virions was 180.43 ± 16.9 nm, with a mean diameter of 136.07 ± 11.28 nm (n = 20). The mean length of the nucleocapsids was 154 ± 20 nm, with a mean diameter of 36 ± 4 nm, mean envelope width was 5.2 ± 0.2 nm (n = 20).

### Complete genome assembly of candidate virus

The alignment of multiple independent assemblies produced a full genome consensus sequence of 107,063 bp (Accession: MK439999). Reassembling the concatenated reads from all samples, after mapping to the candidate consensus sequence, increased coverage to an average of 400.50× (SD: 65.16). The assembled contig of 107,063 bp is concordant with the size of other known nudivirus genomes, as is the estimated GC content of 35.34% (Table 1). REAPR detected no errors or breaks in the assembled genome. PCR confirmation and sequencing of reduced coverage areas revealed the presence of repeating units, which sometimes varied in copy number between independent samples. Sanger reads sequenced from three separate samples confirmed correct assembled sequence.

### Tandem repeats associated with viral replication

**Figure 3:**
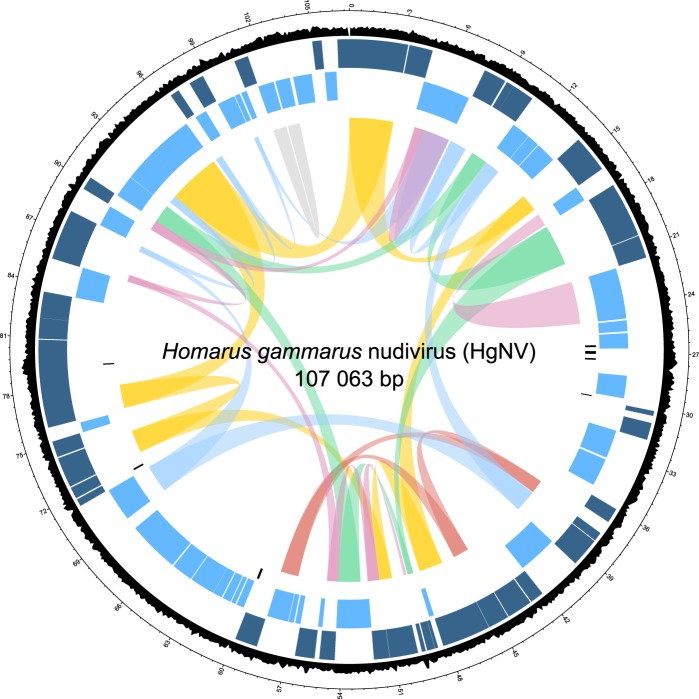
HgNV circular genome plot. Visual representation of HgNV layout scaled to the complete 107 063 bp contiguous sequence. Outermost track shows GC content (%) across complete sequence. Dark blue track displays gene predictions localised to the forward strand, whereas light blue displays those on the reverse. The innermost track depicts direct repeat regions. Links highlight genes involved in similar functions; yellow - DNA replication and repair, red - nucleotide metabolism, green – RNA transcription, pale blue – per os infectivity, pink – packaging and assembly, and grey – apoptosis inhibition.

The HgNV genome does not contain any A/T-rich, palindromic, homologous regions (hrs) that are known to support the origin of replication in baculoviruses and play important roles in viral transcription^25,26^. However, seven direct repeats (drs), ranging from 58.8 to 188 bp were detected (Table 2), two of which fall within protein coding regions. *Eco*RI centres or significant palindromic regions, both typical of hrs, were not detected within these repeating regions. However, dr1-dr4 are clustered within 3.3% of the entire genome; a region of 3,531 bp (Fig. 3). A cluster of drs also appear within the PmNV genome^12^.

**Table 2:** Direct repeat predictions within the HgNV genome.

ID	Position		Consensus Repeat Size (bp)	Copy Number	dr Size (bp)	Percent Identity (%)	Consensus pattern
	Start	End					
dr1	26450	26573	27	4.6	124.2	100	GGAAGCTACACTGGTATTAGATGTAGC
dr2	26856	27043	20	9.4	188	100	GAGCTGAGTTAGTACTGCTG
dr3	27351	27453	36	2.9	104.4	97	CTTATCATGAGAGATTGCCCGGCCACCTGCAGTGGT
dr4	29923	29981	21	2.8	58.8	100	TGTTGATTTTGGATTGTATTG
dr5	59936	60081	32	4.6	147.2	100	TATGACTGATTCTCTGATATATGTACTGTGAT
dr6	71612	71718	51	2.1	107.1	96	CATCGACATCGGAACGATCACCAGAGATTCCACACATACCAACACCCCCAC
dr7	79269	79344	36	2.1	75.6	97	CCACCACCAATGTCCGAAGCCACACTCACTCCACCA

dr = direct repeat. Tandem repeat alignment score of >100.

### Open reading frame (ORF) prediction and genome annotation

Prokka predicted 101 protein coding regions in the HgNV genome. FGenesV0 and GeneMarkerS predicted 103 and 89 ORFs, respectively. Ninety-seven ORFs were supported by two or more programs and were distributed evenly across both strands (Fig. 3, Table 3); 49 on the plus strand and 48 on the minus. The gene density of the HgNV genome was estimated to be 1.10 per kb and 69% of ORFs aligned most closely with predicted genes from the PmNV genome.

**Table 3:** Supported open reading frame annotations of the HgNV genome.

ORF	Strand	Position		Promoter motif	Best BLAST hit		Key HMM/InterPro feature/GO term
		Start	End		Description	%	
01	+	1	3249	TATA, E1, L	**DNA polymerase**	48	DNA_pol_B
02	+	3325	4623	TATA, L	**methyltransferase**	36	FtsJ, TM
03	−	4624	4854	TATA	**PmNV_007**	57	TM, Chondroitin AC/alginate lyase
04	−	4857	5504	L	**Ac92-like protein**	39	Evr1_Alr
05	−	5488	7548	TATA	**Vp91**	38	CMB_14*, TM
06	+	7689	8948	TATA, L	**ODV-E56**	52	Baculo_E56*, TM, Chondroitin AC/alginate lyase
07	+	9062	9487	E1, L	**PmNV_012**	34	
08	+	9500	10717	L	**P47**	39	DNA-directed 5′-3′ RNA polymerase activity
09	−	10735	11868	−	**Pif-2**	58	PIF2, TM
10	−	11900	12652	TATA, L	HZV_115-like protein	29	HAD-like superfamily; P-loop containing nucleoside triphosphate hydrolase
11	−	12706	13965	TATA	**PmNV_018**	24	
12	+	14103	14459	TATA, L	PmNV_019	30	
13	+	14432	15709	TATA, L	**PmNV_020**	40	DNA repair
14	+	15675	16163	TATA, L	**PmNV_021**	40	
15	−	16171	17094	TATA	**Vp39/31 k**	33	
16	+	17244	20309	E1	**LEF-8**	49	DNA-directed 5′-3′ RNA polymerase
17	+	20362	21618	TATA, L	**P51**	41	
18	−	21707	24835	TATA, L	**PmNV_025**	34	Protein AC81, baculovirus
19	−	24952	25623		**PREDICTED: E3 ubiquitin-protein ligase TRIM39-like**	34	zf-RING_UBOX, metal ion binding, acid-amino acid ligase activity
20	−	25742	26152				FSA_C, SP, TM
21	−	28358	29749	TATA			
22	+	30149	30418	E1, L			
23	+	30822	31772	E1, L, HzNV-1	**serine/threonine protein kinase**	30	Pkinase
24	−	31787	33529	TATA, L	**ODV-E66**	33	Chondroitin AC/alginate lyase, SP, TM
25	−	33662	35383	TATA, E1, L	**ODV-E66**	38	Chondroitin AC/alginate lyase, TM
26	+	35948	36772	L	dihydroxy-acid dehydratase	29	EF-hand domain pair
27	+	37022	38011	-	**guanosine monophosphate kinase**	41	Phosphorylation, kinase activity
28	+	38069	39673	TATA	**PIF-1**	47	PIF, TM
29	−	39670	40035	−	PmNV_040	30	Chondroitin AC/alginate lyase
30	−	40016	42100	TATA, L	**PmNV_042**	30	ERV/ALR sulfhydryl oxidase domain superfamily
31	+	42114	42866	L	**hypothetical protein**	40	MqsR_toxin
32	+	42943	43623	TATA	**PmNV_044**	40	
33	+	43620	44348	TATA, L	**PmV-like protein**	26	
34	+	44405	45574	TATA, L	**p-loop NTPase**	41	P-loop containing nucleoside triphosphate hydrolase
35	+	45601	46521	TATA, L	**PmNV_047**	46	
36	+	46527	48293	TATA	**PmNV_048**	36	DNA_ligase_A_M
37	−	48281	48577	TATA, L	**hypothetical protein**	33	
38	+	48594	48761	TATA	PmNV_051	31	TM
39	+	48781	49212	TATA	hypothetical protein	53	Baculo_LEF5_C
40	+	49299	49508	L			Ribonuclease H-like superfamily
41	+	49733	50383	TATA, L	**PmNV_054**	33	
42	+	50380	51297	TATA	**integrase**	50	Phage_integrase
43	+	51319	52179	E1, L	**VLF-1**	41	Phage_integrase
44	−	52185	52547	E1, L	surface-associated interspersed protein (SURFIN)	24	
45	−	52492	52713	L			
46	−	52713	54332	TATA	**LEF-9**	54	RNA_pol_Rpb1_2
47	+	54340	55185	−	**38 K protein**	42	NIF
48	−	55182	55448	TATA			
49	+	55450	56160	TATA, L	**PmNV_061**	41	YopH_N*
50	+	56153	56533	TATA, L	**PmNV_062**	32	TM
51	−	56543	56863	TATA, L	PmNV_063	34	
52	−	56956	57270	TATA, L			TM
53	−	57311	58627	−	**p-loop NTPase**	34	
54	+	58684	59928	−	**PmNV_066**	38	Chondroitin AC/alginate lyase
55	−	60168	60629	TATA			membrane
56	−	60717	60980	TATA			Per os infectivity factor
57	−	61046	61567	E1	PmNV_067	32	
58	−	61649	62212	TATA, L	**PmNV_068**	28	
59	−	62233	63204	TATA, L	**PmNV_069**	45	
60	−	63197	63781	L	**PmNV_070**	30	
61	−	63840	65159	TATA, L	**PmNV_071**	26	
62	−	65256	68456	TATA	Conserved hypothetical protein*	41	Ribonuclease H-like superfamily, ubiquitin-protein transferase activity
63	−	69215	71284	TATA, L, HzNV-1	**P74**	55	Baculo_p74_N, Baculo_p74, TM
64	+	71332	71727	TATA, E1			Zinc finger, RING-type, TM
65	+	71814	72452	TATA			
66	+	72535	74214	L	**helicase 2**	48	PIF1, Viral_helicase1*, P-loop containing nucleoside triphosphate hydrolase
67	+	74299	75180	TATA, L	PmNV_077	24	Zinc finger, RING-type
68	−	75187	75795	L	**PmNV_078**	38	Ribonuclease H superfamily
69	+	75980	77722	−	**helicase 2**	41	Viral helicase1*, S-adenosyl-L-methionine-dependent methyltransferase
70	+	77724	80855	L			P-loop containing nucleoside triphosphate hydrolase
71	+	80914	82050	E1, L	**PREDICTED: uncharacterized protein LOC108666550**	36	S-adenosyl-L-methionine-dependent methyltransferase
72	+	82064	82945	L	**PmNV_082**	39	
73	+	82939	83511	E1, L			
74	−	83508	85004	TATA, L	PmNV_084	27	
75	−	84998	85399	−	**PmNV_085**	34	
76	+	85386	85892	TATA, L	**Ac81-like protein**	60	Ac81, TM
77	+	85867	87768	TATA, E1, L	**PmNV_087**	34	
78	+	87755	88186	−	**Ac68-like protein**	46	TM
79	−	88212	89504	TATA, L	**PmNV_089**	32	
80	+	89589	90314	L	**VLF-1**	42	DNA binding, DNA integration, DNA recombination
81	−	90317	91672	TATA, E1	**LEF-4**	38	regulation of transcription
82	−	91700	92032	E1, L	PmNV_092	55	
83	−	92043	92606	TATA, E1, L	**PIF-3**	50	PIF3, TM
84	−	92599	96429	TATA, L	**helicase**	39	helicase activity
85	+	96641	97144	TATA	**ODV-E28**	53	ThrE*
86	−	97106	97846	TATA, L	**PmNV_097**	33	
87	+	97864	98697	TATA	**esterase**	54	Alpha/Beta hydrolase fold, TM
88	−	98701	99804	TATA, E1, L	**PmNV_099**	40	ERV/ALR sulfhydryl oxidase domain superfamily
89	−	99855	100166	TATA	**11 K virion structural protein**	55	TM
90	−	100274	100618	E1			
91	+	100608	101390	−	**PmNV_102**	34	
92	−	101387	102367	TATA	**death-associated inhibitor of apoptosis 1**	30	BIR, zf-C3HC4_3
93	−	102481	103380	TATA, L	**PREDICTED: baculoviral IAP repeat-containing protein 7-like**	31	Zf-C3HC*, BIR
94	−	103599	104747	TATA, L			SP, TM
95	+	105021	105533	TATA, E1, L			RNA polymerase, beta subunit, conserved site
96	−	105557	106270	TATA, L	**PmNV_107**	51	
97	+	106404	107060	TATA, E1, L	**PmNV_108**	39	Membrane

BLAST annotations with an E-value equal or greater than 1 are not shown. Annotations with an E value > x10^10^ are highlighted in bold. *Pfam annotations with an E-value less than 1. SP = signal peptide, TM = transmembrane domain.

**Figure 4:**
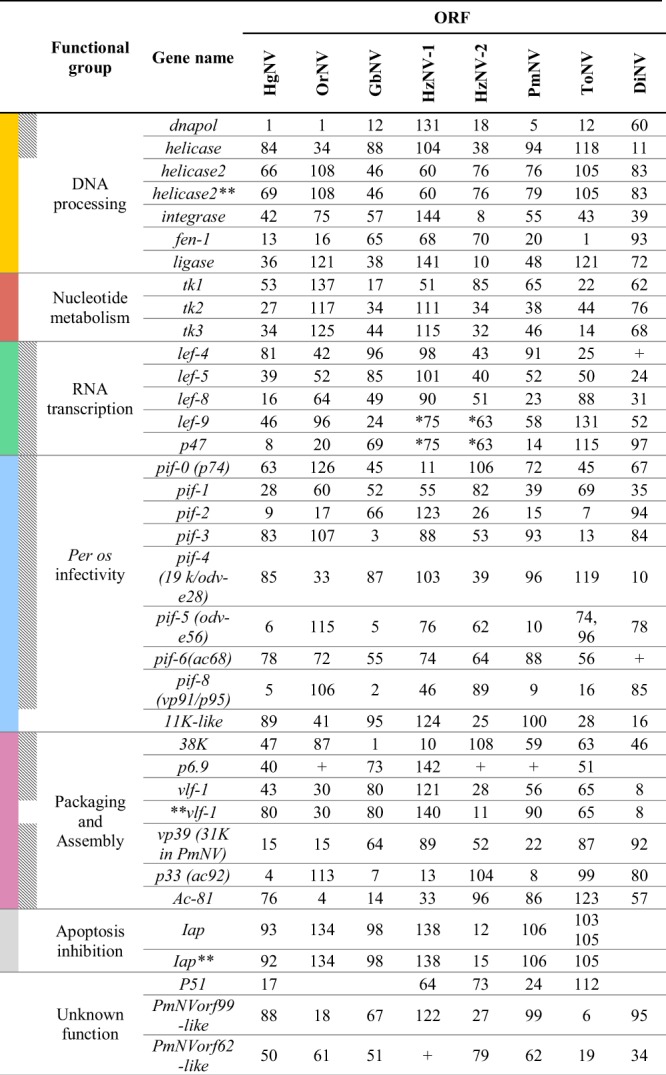
HgNV homologs to conserved nudivirus sequences. Colours as in Fig. 3. *Fused to a single gene. **Multiple copy number. Shaded cells of second column indicate ‘core nudivirus genes’ shared with the *Baculoviridae*. + Reported present.

The exact number of genes conserved across all the nudiviruses is somewhat unclear. However, re-analyses of all sequenced nudivirus genomes revealed a set of 21 core genes conserved between baculoviruses and nudiviruses^13^. The core genes were typically grouped into one of five functional groups: DNA processing, RNA transcription, *per os* infectivity, package and assembly and conserved genes of unknown function. The HgNV genome contained 7 genes involved in DNA processing; *DNA polymerase*, *helicase*, two copies of *helicase2*, *integrase*, *fen-1* and *ligase*. Gene predictions similar to three of the four thymidine kinase (*tk*) genes involved in nucleotide metabolism were also found. All five core baculovirus/nudivirus genes involved in RNA transcription were found; *p47*, *lef-4 (late expression factor)*, *lef-5*, *lef-8* and *lef-9*. As were 8 genes involved in *per os* infectivity: *pif-0* (*p74*), *pif-1*, *pif-2*, *pif-3*, *pif-4* (*19 k/odv-e28*), *pif-5* (*odv-e56*) and *pif-6* (*ac68*) and *pif-8 (vp91/p95)*. The *11K*-like gene was also found^27^. HgNV contained less than half of the genes encoding packaging, assembly and release processes conserved amongst the *Baculoviridae*. These include a *38* *k* gene, *p6*.*9*, two copies of *vlf-1*, *vp39*, *p33* (*ac92*) and *ac-81*. Similar to PmNV, HgNV also possessed 2 copies of the *Iap* genes involved in apoptosis inhibition. Furthermore, HgNV encoded sequences similar to PmNVorf99 and PmNVorf62, reported to be common in nudiviruses (Fig. 4). Other genes typically common to baculoviruses, including *methyltransferase* and two neighbouring copies of *odv*-*e66*, were also found in the HgNV genome.

### Promotor regions preceding ORF predictions

Analysis of the 300 bp region upstream of each ORF start codon revealed the presence of promotor motifs in all but 12 of the predicted coding regions (Table 3). Early promotors defined by a TATA box with or without E1 motifs were predicted for 73 ORFs. No E2 motifs were detected. Late (L) promoters were predicted for 59 ORFs with HzNV-1 specific late promoters predicted for two coding regions; HgNV_ORF23, exhibiting a protein kinase structural domain and HgNV_ORF63 coding for the *p74* gene. A combination of early and late promoters were predicted to precede 47 potential coding regions.

### Phylogenetic characterisation of HgNV

**Figure 5:**
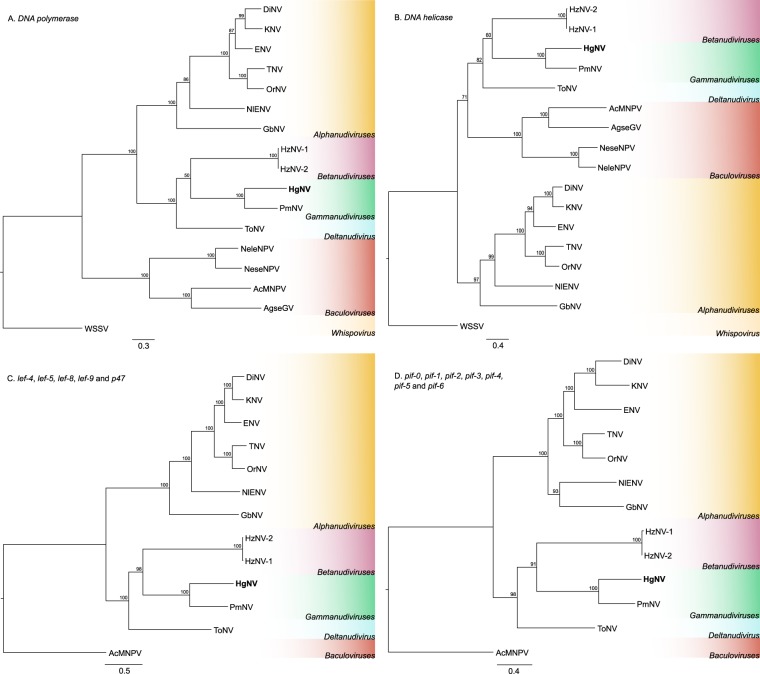
Single and multigene phylogenies of known nudiviruses. Maximum Likelihood analyses of nudivirus phylogeny, including whispovirus and baculovirus outgroups. Node labels indicate bootstrap support (%). (**A**) – Single gene phylogeny of *DNA polymerase*. (**B**) – Single gene phylogeny of *DNA helicase*. (**C**) – Multigene phylogeny of late expression factors (*lef-4*, *lef-5*, *lef-8*, *lef-9* and *p47*). (**D**) – Multigene phylogeny of *per os* infectivity genes (*pif-0*, *pif-1*, *pif-2*, *pif-3*, *pif-4*, *pif-5* and *pif-6*). DiNV – *Drosophila innubila* nudivirus, KNV – Kallithea virus *(D*. *melanogaster*), ENV – Esparto virus (*D*. *melanogaster*), TNV – Tomelloso virus (*D*. *melanogaster*), OrNV – *Oryctes rhinoceros* nudivirus, NleNV – *Nilaparvata lugens* endogenous nudivirus, GbNV – *Gryllus bimaculatus*, HzNV-1 – *Heliothis zea* nudivirus 1, HzNV-2 – *Heliocoverpa* (syn. *Heliothis*) *zea* nudivirus 2, HgNV – *Homarus gammarus* nudivirus, PmNV – *Penaeus monodon* nudivirus, ToNV - *Tipula oleracea* nudivirus, NeleNPV – *Neodipirion lecontei* nucleopolyhedrovirus, NeseNPV – *Neodipirion sertifer* nucleopolyhedrovirus, AcMNPV – *Autographa californica* multiple nucleopolyhedrovirus, AgseGV – *Agrotis segetum* granulovirus, and WSSV – white spot syndrome virus. Coloured clade groupings refer to proposed genera: yellow – *Alphanudiviruses*, pink – *Betanudiviruses*, green – *Gammanudiviruses*, blue – *Deltanudiviruses*, red – *Baculoviruses*, cream – *Whispovirus*.

Single gene phylogenies using the *DNA polymerase* and *helicase* genes showed contrasting positioning of the *Nudivirus* and *Baculovirus* clades, however both grouped HgNV with PmNV, together with ToNV, HzNV-1 and HzNV-2 (Fig. 5A,B). Multigene analyses of all shared genes involved in transcription (*lef*-4, *lef* -5, *lef* -8, *lef* -9 and p47) and *per os* infectivity (*pif-0*, *pif*-1, *pif*-2, *pif*-3, *pif*-4, *pif*-5, *pif*-6) within the nudiviruses very robustly supported this grouping, with Maximum Likelihood (ML) bootstrap values of 100% and 98% respectively (Fig. 5C,D).

## Discussion

Here we provide the first description of a naturally-occurring virus infection of nephropid lobsters. The virus, *Homarus gammarus* nudivirus (HgNV) is a new species within the family *Nudiviridae*; a group of dsDNA viruses that infect arthropod (mainly insect) hosts. Histopathology and ultrastructure of HgNV is similar to numerous other bacilliform viruses described to infect Crustacea, wherein viral replication within the host nucleus displaces host chromatin and results in aberrant, hypertrophied nuclei, visible in routine histological preparations. In many cases, infected epithelial cells are sloughed off the basement membrane of the tubule into the lumen, for excretion via the faeces. It is important to consider that intranuclear inclusions may also be indicative of other pathogens as this may explain discrepancies in prevalence when comparing PCR and histology data. Furthermore, digestive tissues are known to contain inhibitors which can impact PCR success^28,29^. However, *in-situ* hybridisation confirms that HgNV is inducing this pathology in infected cells (Fig. 1C,D).

Comprehensive genome analysis of infected lobsters revealed that HgNV is most closely related to PmNV, a virus infecting the black tiger shrimp, *Penaeus monodon*. However, despite a high degree of conservation in gene order, the percentage identity of HgNV gene predictions to known annotations was fairly low, averaging just over 38%. Our Maximum Likelihood phylogenies were concordant with previously published trees, which indicate that PmNV may belong to a separate genus within the *Nudiviridae*; the *Gammanudiviruses*^12^. Our phylogenetic analyses show that HgNV also belonged to this clade and, together with PmNV, could represent a radiation of nudiviruses infecting diverse aquatic crustacean taxa. Based on the long branch lengths of the neighbouring lineages, it is likely that ToNV and both HzNV-1 and HzNV-2 belong to separate genera; provisionally referred to as *Deltanudivirus* and *Betanudivirus* respectively (Fig. 5). We also show that the newly sequenced *Drosophila* nudiviruses belong to the *Alphanudivirus* clade and present the most substantial nudivirus phylogeny to date. The multigene phylogeny of the late expression factors provides bootstrap support of 100% in all but one node (98%) (Fig. 5C).

Nudiviruses contain a distinct repertoire of genes involved in DNA processing, compared to the baculoviruses. Two *lef* genes and an *alk-exo* gene are absent in HgNV. *Lef-1* has been shown to be associated with DNA primase activity which aids in polymerisation, and *lef-2* is thought to stabilize the binding of *lef-1* to the DNA molecule^30^, amplifying replication^31^. HgNV contains two copies of the *helicase-2* gene which are also found in the PmNV genome (HgNV_ORF66, HgNV_ORF69). Both genes are predicted to contain features characteristic of helicase activity. An *integrase* gene is also common to all sequenced nudiviruses and is represented by HgNV_ORF42, which contains the phase_integrase domain involved in the integration of viral DNA into the host genome. This is noted to facilitate persistent infection of HzNV-1 in its host^32^.

Of the five core genes involved in RNA transcription, *P47* (HgNV_ORF08) encodes a viral transcription regulator, involved in late stage infections, reported to make up one of the four subunits of RNA polymerase, whereas the four remaining *lef* genes are thought to regulate late and very late gene expression, and are named to reflect their synthesis during infection. In contrast, early gene expression is instead initiated by host-derived RNA polymerase^33^.

Baculovirus life cycles are typically split between occlusion-derived virus (ODV) and budded virus (BV) stages. *Per os* infectivity genes, conserved within the HgNV genome, are required for the infectivity of ODVs that facilitate the transmission of viral particles from one host to the next, whereas BVs spread virions to neighbouring cells. *Pif-0*,*1 and* 2 encode envelope proteins vital for oral infection and are thought to bind virions to the midgut cells^34^. However, *pif-3* does not affect midgut binding. *Pif-3* is instead hypothesised to interact with the viral cytoskeleton and play a role in translocation of the capsid^35^. It is believed that *pif-1*, *2*, 3 and 4 form a multimolecular protein complex that is vital for oral infectivity, with other *pif* genes associating with the core complex at a lower affinity^36^. *Pif-7¸*originally described as the ODV-envelope protein Ac110, also associates with the complex but was not found in the HgNV genome. However, Pif-8, previously described as the structural protein vp91/p95, was detected (HgNV_ORF05) and is predicted to contain chitin-binding peritrophin-A domains. The peritophic membrane surrounds the food bolus and lines the gut of most crustaceans and serves to separate large particulate matter from the epithelial cells and limits the penetration of microbes^37^. HgNV also encodes a homolog to an 11 K protein noted to enhance oral infection. These 11 K proteins typically contain binding motifs common to mucins, peritrophins and chitinases and could facilitate midgut binding, typically occurring after the alkaline dissolution of the occlusion lattice^38^.

The reduction in genes encoding packaging, assembly and release is likely a reflection of the lack of occlusion bodies in the transmission strategy adopted by the *Nudiviridae*. HgNV_ORF47 encodes a *38K*-like gene which mediates the dephosphorylation of the C terminus of the *p6*.*9* gene; a gene responsible for the encapsulation of the viral genome. Although not detected through BLAST alignment, likely a result of its highly repetitive sequence, HgNV_ORF40 was identified as the *p6*.*9* gene after alignment with other annotated sequences. Furthermore, alignment of the hypothesised PmNV coding region of the *p6*.*9* gene to the HgNV genome corresponds to a region within the HgNV_ORF40 predicted ORF. Similarly to PmNV, HgNV shares two separate sequence homologs to the *vlf-1* gene (HgNV_ORF43, HgNV_ORF80), responsible for very late gene expression and proper formation of the nucleocapsid^39^. HgNV also encodes a second major capsid protein: *p33/ac92* (HgNV_ORF04). HgNV_ORF04 reports an Erv1_Alr feature, belonging to a family of sulfhydryl oxidases. Prior analyses and purification of ac92 suggests it is a flavin adenine dinucleotide (FAD) containing sulfhydryl oxidase^40^. The major viral capsid protein *vp39* is thought to be a core baculovirus/nudivirus gene and was reportedly mislabelled as a *31K*-like structural protein in PmNV (ORF_022). HgNV_ORF15 shares 33% identity across 99% of the PmNV_022 sequence and 21% identity across 91% of HzNV-1 ORF89 and HzNv-2 ORF52, also annotated in GenBank as 31K-like proteins. However, the similarity of HgNV_ORF15 with the *vp39* genes of other nudiviruses is much lower. Protein domains could not be predicted to aid in its clarification.

The PmNV_099-like coding region is also shared amongst the nudiviruses and HgNV_ORF88 indeed shares 40% sequence identity with PmNV_099, which is described as ‘microtubule-associated-like’^12,13^. This gene could play a role in the rearrangement of the host nucleus during viral replication, whereby host chromatin is translocated to the inner nuclear membrane, a process thought to be dependent on viral interaction with host tubulin^35^. Much like *ac92*, HgNV_ORF88 is also predicted to contain the ERV/ALR sulfhydryl oxidase feature which can play a role in virion assembly by catalysing disulphide bond formation between cysteine residues^41^. In regard to HgNV gene predictions found in the baculoviruses, HgNV_ORF02 shares 35.86% identity to a methyltransferase annotated in the PmNV genome, hypothesised to be involved in viral RNA capping^42^. As is the case with PmNV, HgNV encodes two neighbouring *odv-e66* predictions responsible for the trafficking of viral proteins during infection^43^. Odv-e66 was also reported as the first chondroitin lyase^44^. Chondroitin is an extracellular matrix polysaccharide and its degradation by pathogenic bacteria facilitates access to the target cell^44^. Chondroitin AC/alginate lyase Interpro features were also identified in HgNV_ORF24 and HgNV_ORF25.

There are 37 core genes reported to be conserved amongst the baculoviruses^45^. Assuming the 31K-like gene is in fact a *vp39* homolog, HgNV encodes all 21 core baculovirus genes proposed to be conserved across the nudiviruses (Fig. 4). Nearly half of HgNV predicted coding regions were preceded by both early and late promoter regions (Table 3), suggesting plasticity in the way HgNV can regulate gene expression. However, as stated by Bezier *et al*.^13^ gene expression chronology should not be generalised to promoter motifs alone. Transcriptome analysis of the baculovirus AcMNPV failed to associate reliable sequence motifs with gene expression patterns^46^.

We did not observe evidence of occlusion body formation within our histological or TEM analyses. Similarly, we did not detect sequence homologs of the *poly/gran* gene, which encodes the protein that forms the structural lattice. Ingestion of occlusion bodies allows passage to the digestive tract, where alkalinity of the gut causes the proteinous lattice to dissolve, releasing the virions within and initiating infection^33^. Much like the baculoviruses, the nudiviruses surrounding HgNV (HzNV-2, ToNV and PmNV) can rely on occlusion bodies to facilitate transmission outside of the host. As it would seem that HgNV does not form occlusion bodies, it begs the question of how viral particles remain viable during horizontal transmission. An alternative infection strategy would be that HgNV persists as a latent virus within the host and its evolution has favoured the maintenance of low virulence, which subsequently translates to an increase in transmission through longer lasting infections, as infection doesn’t incapacitate the host. Viruses infecting cells of the digestive tract sloughed out of the animal may remain viable until the degradation of the excreted cell. The ingestion of faeces may therefore serve as possible route of transmission for HgNV^47^. Latency within the host is a shared strategy true of several other shrimp viruses and supported by field data relating to the infection of the marine shrimp *Crangon crangon* by a putative nudivirus, where prevalence can reach 100% in wild populations^23,48,49^. Alternatively, HgNV may persist in reservoirs outside of its currently known host.

Due to the short life-cycle and seasonal development of their host, insect viruses, like the baculoviruses, are unable rely on either latency or reservoir strategies^35^. Therefore, resistant occlusion bodies would ensure viability outside of the host and facilitate transmission to the next. However, compared to penaeid shrimp, lobsters have very long life-cycles (decades). As such, a virus infecting these animals *can* rely on latency and is not required to survive long periods within the environment. In further support of this theory, occlusion-derived viruses infecting the insect midgut rely on occlusion body-associated enhancins, or similar factors, that digest the chitin lining of the midgut and facilitate entry^35^. However, the hepatopancreas of the lobster is not chitinous^50^. Therefore, HgNV would not depend on OB-associated proteases to gain entry into hepatopancreatic cells. Slack and Arif (2007) hypothesise that baculovirus ancestors were not occluded and instead relied on alkaline proteolytic activation during infection. It is hypothesised that contrasting ecological niches occupied by the insect host life cycle, limit baculoviruses infection to larval stages^15^. Therefore, occlusion body-facilitated horizontal transmission is vital for its longevity within the environment. The non-occluded nudiviruses, however, have demonstrated their ability to infect adult life stages. Therefore evolutionary maintenance of occlusion body transmission offers little benefit over vertical transmission or latency within the aging host^15^.

The expanding diversity of the *Nudiviridae* suggests that lack of occlusion alone is not a distinguishing characteristic of these viruses; several occlude prior to horizontal transmission whereas others do not. It is therefore likely that other characteristics of the genome underlie the separation of the group from the baculoviruses. Little is known about the nudivirus lifecycle and so this, and the means by which they gain entry into the host cell and cause infection, may also serve as discernible features of the proposed genus.

We did not observe any accompanying clinical signs in HgNV-infected individuals. Evidence suggests a persistent asymptomatic virus may even offer benefit to the individuals within an infected population. Invertebrates lack a typical adaptive immune system, however, host cells infected with latent Hz-1 virus (HzNV-1) are resistant to a more virulent infection of the same virus via homologous interference^32^. Nevertheless, despite widespread latency within the *Nudiviridae*, many cause delayed development and eventually death^13^. Whether HgNV has an effect on growth development or mortality of the European lobster remains to be shown. Furthermore environmental and/or physiological stimuli can result in massive viral amplification which give even low virulent viruses the potential to cause mass mortalities within a population^49^. This may be of particular importance as invertebrate aquaculture grows in popularity. The increased prevalence of HgNV in hatchery vs SBCC lobsters suggests either that conditions within SBCC are not conducive to high prevalence (e.g. lower transmission potential) or, that lobsters infected with HgNV have higher mortality during early deployment and thus are not present at later stage sampling points. However, in relation to the latter, given that early mortality in SBCC and hatchery populations did not differ (data not shown), HgNV as a driver of mortality in SBCCs appears unlikely. It should be noted that recirculating systems likely serve as drivers for increased prevalence in older hatchery-reared stocks (52–104 weeks post deployment controls) and juvenile lobsters are not typically on-grown in hatchery environments for such extended periods. Further work on the role of HgNV in early life stage growth and mortality is now required.

## Materials and Methods

### Experimental design and sample collection

Over the period of July 2016 to April 2017, 14,507 hatchery-reared juvenile lobsters were deployed in SBCCs anchored off the coast of Cornwall (St. Austell Bay 50° 18.956 N, 4°44.063 W). The majority of those deployments (10,987 animals), including those used in the current study, occurred in the summer of 2016. Routine sampling (3, 6, 28, 39, 52 and 104 weeks post deployment) was carried out to monitor the incidence of disease in SBCC populations. In total, 1,698 animals were sampled over the 2-year period. Another set of lobsters were retained within the National Lobster Hatchery, Padstow UK, and sampled at the same time points, over this period. Carapace length and survival were measured at each time point. Upon sampling, larger animals (39–104 weeks post deployment) were anaesthetised on ice for several minutes prior to bisection through the dorsal line. One half was fixed in Davidson’s Seawater fixative for histological processing, the other fixed in molecular grade ethanol for sequence analysis. A small piece of hepatopancreas was removed and fixed for transmission election microscopy. Smaller animals (0–28 weeks post deployment) were fixed whole and underwent separate analyses.

Six juvenile lobsters displaying pronounced histopathology associated with viral infection were selected from hatchery and SBCC settings, allowing for comparative molecular and transmission election microscopy analyses. Five of the six animals had spent one to two years growing in controlled hatchery raceways. The remaining individual had spent 52 weeks in SBCC in the open sea.

### Histopathology

Bisected lobsters were placed in to histological cassettes and fixed in Davidson’s Seawater Fixative for 24–48 h before transfer to 70% industrial denatured alcohol (IDA). Cassettes were processed using a Leica Peloris Rapid Tissue Processor and subsequently embedded in paraffin wax. Histological sections were cut using a rotary microtome set at 3 µm thickness, adhered to glass slides and stained using a standard haematoxylin and eosin (H&E) protocol. Slides were examined using a Nikon Eclipse light microscope and NIS imaging software at the International Centre of Excellence for Aquatic Animal Health at the Cefas Laboratory, Weymouth, UK.

### Transmission electron microscopy

Hepatopancreas samples were fixed in 2.5% glutaraldehyde in 0.1 M sodium cacodylate buffer (pH 7.4) and later rinsed in 0.1 M sodium cacodylate buffer prior to processing. Post-fixation was carried out in 1% osmium tetroxide/0.1 M sodium cacodylate buffer for 1 h. Tissues were washed in three changes of 0.01 M sodium cacodylate buffer and were subsequently dehydrated through a graded acetone series before embedding in Agar 100 epoxy resin (Agar Scientific, Agar 100 pre-mix kit medium). Embedded tissues were polymerised overnight at 60 °C. Semi-thin (1–2 µm) sections were cut and stained with Toluidine blue for viewing with a light microscope to identify suitable target areas. Ultra-thin sections (70–90 µM) of targeted areas were mounted on uncoated copper grids and stained with 2% aqueous uranyl acetate and Reynold’s lead citrate^51^. Grids were examined using a JEOL JEM1400 transmission electron microscope and digital images captured using an AMT XR80 camera and AMT V602 software.

### DNA extraction and sequencing

DNA for genomic reconstruction was extracted using the CTAB/phenol:chloroform extraction protocol as described in Holt *et al*.^52^. DNA for HgNV screens of HP tissue was extracted using the EZ1 Advanced XL and DNA Tissue Kit (Qiagen). Extracted DNA was cleaned with polyethylene-glycol (PEG) precipitation and submitted to the sequencing service at the University of Exeter, UK for shotgun library preparation using the TruSeq DNA PCR-Free Library Prep Kit. Pooled libraries underwent high-throughput sequencing using an Illumina Miseq (2 × 300 bp).

### Sequence analysis

The raw Illumina paired-end sequence reads generated were quality-checked using FastQC v0.11.4^53^ and subsequently trimmed to remove adapter sequences and low-quality bases using Trimmomatic v0.36^54^. Sequence reads were error-corrected and digitally normalised using bbnorm (part of BBMap v38.22)^55^ and reads of each sample were assembled individually with Unicycler v0.4.7 (using default parameters and–no_correct)^56^. Quality-trimmed paired reads from individual samples were also assembled *de novo* using the A5-miseq assembly pipeline^57^. Contigs representing putative HgNV were aligned using progressiveMauve (build date Jun 26 2018)^58^ in order to obtain a consensus sequence. In order to identify viral contigs, Prokka^59^ was used to identify protein-coding regions spanning the assembled contigs and these were subsequently annotated using the BLASTp algorithm of Diamond v0.7.9^60^ and the full NCBI non-redundant (nr) protein database (20170515). Sequences representing dsDNA viruses were identified by visualising the Diamond output in MEGAN6 Community Edition v6.5.5^61^ and corresponding contig sequences were extracted. Paired reads from all samples were subsequently mapped to the candidate genome contigs using BWA-MEM 0.7.12-r1039^62^ and visualised with the Integrative Genomics Viewer (IGV) v2.3.68^63^ Assembly quality and accuracy were assessed with QualiMap v2.0^64^ and REAPR (version 1.0.18)^65^. Predicted open reading frames (ORFs) were identified using three different tools, including Prokka, FGenesV0 (softberry.com) and GeneMarkS^66^ (amino acid size of 50, circular genome). ORFs that were supported by two or more programs were analysed further. In cases where multiple ORFs were predicted to overlap, the largest sequence was chosen. Supported ORFs were annotated using NCBI BLASTp and the full NCBI nr protein sequence database (20180803).

Tandem repeats within the final assembled genome were identified using the tandem repeats finder using default parameters^67^. Repetitive regions with an alignment score of 100 or more were further analysed for palindromic sequences using the MEME program and a minimal size of 20 bp^68^. Promoter sequences were located within 300 nucleotides upstream of ORF start codon predictions using the Geneious software package v.11.1.4^69^. Early promoters contain TATA[AT][AT][AT] sequences. TATA boxes may also associate with CA[TG]T (E1) or CGTGC (E2) 20–40 nucleotides downstream. The baculovirus late promotor (L) corresponding to [ATG]TAAG and the HzNV-1-specific late promoter (HL) were also queried using the sequence TTATAGTAT.

A circular map of the HgNV genome was plotted using shinyCircos^70^. The assembled HgNV genome and corresponding ORF predictions are deposited in GenBank under the genome accession number MK439999.

### Molecular confirmation of genome assembly

To resolve ambiguous regions of the genome assembly, primers were designed that span areas of lower coverage and INDEL queries. PCR amplification was performed in 50 µL volumes using 10 µL of Promega 5X Green GoTaq Flexi Buffer, 5 µL of MgCl_2_, 0.5 µL of each primer (Final concentration; 1 µM), 0.5 µL of DNTPs, 0.25 µL of GoTaq DNA Polymerase, 30.75 µL of molecular grade water and 2.5 µL of template DNA. Initial denaturation was carried out at 94 °C for 2 min. This was followed by 30 PCR cycles of denaturation at 94 °C for 1 min, annealing at 60 °C for 1 min and extension at 72 °C for 1 min, followed by a final extension at 72 °C for 5 min. Sequenced amplicons were aligned to the candidate genome using the multiple sequence alignment program (MAFFT Version 7)^71^ and assembly was assessed across query regions.

Diagnostic primers were constructed from the alignment of *DNA polymerase* gene sequences. HgNV_DNAPol_F1: 5′ACTTGAAGCTGTGCGTGACT 3′ and HgNV_DNAPol_R1: 5′ TGTATGTCTTGCGGCCCATT 3′ produce an amplicon of 383 bp and only anneal to HgNV when queried with Primer-BLAST and the nr database. PCR conditions were as above. Amplicons were cleaned with the GeneJET PCR Purification Kit (Thermo, US) and sequenced via the Eurofins TubeSeq service. HgNV_DNAPol primers were tested on 150 SBCC and 12 hatchery lobsters (sampled at 52 weeks post-deployment). Shrimp tissues infected with WSSV and a putative nudivirus were tested as negative controls and did not amplify.

### Phylogenetic tree construction

Homologous target genes were aligned using the multiple sequence alignment program MAFFT Version 7^71^; and the E-INS-I iterative refinement method. Multigene alignments were constructed by concatenating gene sequences prior to alignment. A maximum likelihood phylogenetic tree inference was constructed using RAxML-HPC BlackBox version 8^72^ on the CIPRES Science Gateway^73^ using a generalised time-reversible (GTR) model with CAT approximation (all parameters estimated from the data).

### *In-situ* hybridisation

An extended HgNV-specific *DNA polymerase* probe which spanned and the HgNV_DNAPol amplicon sequence was designed to optimise the hybridisation protocol. HgNV_DNAPol_ISH_1838f: 5′ AGATTGAGCAGAGTGTAGCCC 3′ and HgNV_DNAPol_ISH_2799R 5′ ACCTTCCGATGATAGTTCTTCC 3′ produce an amplicon of 961 bp. *In-situ* hybridisation of the extended HgNV probe was carried out following the protocol described by Bojko *et al*. 2019^74^ using a 2X washing buffer (20X SSC, 0.2% BSA, 6 M Urea). However, NBT/NCIP incubation was limited to 15 minutes and slides were instead counterstained with 0.1% Fast Green solution.

## Data Availability

Sequences have been deposited in GenBank under the BioProject PRJNA516791.
